# Diagnostic performance of conventional MRI using T1W and T2W for primary lymph node staging in intermediate- and high-risk prostate cancer patients prior to pelvic lymph node dissection

**DOI:** 10.1007/s00261-025-05073-w

**Published:** 2025-06-27

**Authors:** Georgios Daouacher, Jessica Carlsson, Nikolaos Voulgarakis, Sofia Papageorgiou, Pär Dahlman, Pernilla Sundqvist, Mauritz Waldén

**Affiliations:** 1 Karlstad Central Hospital, Karlstad, Sweden; 2 Örebro University, Örebro, Sweden; 3 Örebro University, Örebro, Sweden; 4 Uppsala University Hospital, Uppsala, Sweden; 5 Värmlands Läns Landsting, Karlstad, Sweden

**Keywords:** Prostate cancer, Lymph node staging, ΜRI

## Abstract

**Purpose:**

To assess the accuracy of conventional MRI with T1- and T2-weighted sequences in detecting lymphatic nodal spread (N1) in intermediate- and high-risk prostate cancer (PCa) patients via morphological criteria alone, extended pelvic lymph node dissection (ePLND) was used as the reference standard.

**Methods:**

This prospective observational study included patients between 2009 and 2016 with intermediate- and high-risk PCa according to the D’Amico criteria and an estimated risk of N1 > 20% on the basis of the Briganti nomogram. All patients underwent MRI prior to ePLND. Interobserver analysis was conducted across three centers.

**Results:**

Ninety-nine men, mean age 67 (5.7 SD), 93% high-risk PCa patients and 39.4% with N1 disease, according to ePLND, were evaluated. The pooled sensitivity of MRI for detecting N1 was 24.6% (95% CI: 16.3–35.1), whereas the pooled specificity was 95% (95% CI: 85.3–98.8). Interobserver agreement was moderate (Fleiss’ κ = 0.56). All readers failed to identify patients with high-volume N1, and the identification of those with a high number of N1 events was inconsistent across readers. The strengths of this study include the high number of N1 cases, with a median of 17 (6–40) harvested lymph nodes per participant. Limitations include the time interval between MRI and ePLND (median of 44 days) and the lack of standardized lymph node evaluation criteria, reflecting real-world clinical practice.

**Conclusion:**

MRI using only T1W and T2W sequences has demonstrated limited effectiveness in lymph node staging for intermediate- and high-risk prostate cancer, even in high-volume metastatic disease. Additionally, interobserver analysis shows only moderate agreement.

## What does this study add?

**Table:** 

This study demonstrated that T1W, which is currently used in prostate MRI protocols for nodal evaluation, and T2W MRI sequences are not effective for evaluating lymph nodes in prostate cancer patients, even when a significant amount of nodal metastasis is present.	

## Clinical relevance

**Table:** 

Given the widespread use of MRI in prostate cancer diagnosis, MRI protocols should be revisited to increase the detection of nodal spread in high-risk patients. Improving detection could facilitate earlier intervention for metastatic disease.	

## Introduction

Accurate clinical staging is essential for prostate cancer (PCa) management prior to treatment counseling. Lymphatic nodal spread (N1) is associated with an increased risk of biochemical recurrence and prostate cancer-specific mortality [1]. According to the European Association of Urology (EAU) guidelines, PCa staging typically relies on bone scintigraphy and conventional cross-sectional imaging modalities such as CT or MRI [2]. However, the gold standard for nodal assessment remains extended pelvic lymph node dissection (ePLND). Despite its accuracy, ePLND is often avoided because of its high cost and associated morbidity [3, 4].

As a result, considerable effort has been made to improve noninvasive imaging for nodal staging. A meta-analysis by Hovels et al., including studies from 1980 to 2003, revealed that CT and MRI have limited sensitivity in detecting N1 disease, particularly in identifying low-volume metastatic disease and normal-sized metastatic lymph nodes [5]. Improved detection has been observed with MRI combined with diffusion-weighted imaging (DWI) [6, 7]. PSMA PET-CT has also shown promising results for nodal staging [8], but its widespread use is limited by high costs and restricted global availability [9].

In contrast, prostate MRI is widely used prior to guided biopsies for PCa detection, primarily via DWI focused on the prostate. However, the Prostate Imaging–Reporting and Data System (PIRADS) version 2.1, which dictates the current prostate MRI protocols, suggests only T1w sequences for nodal assessment and provides only basic morphological guidelines for nodal evaluation [10]. Notably, no study has evaluated the baseline accuracy of conventional MRI without DWI for N1 detection in PCa patients on the basis solely of morphological criteria validated by ePLND.

This study aims to evaluate the ability of nonfunctional MRI to detect N1 disease in patients with intermediate- and high-risk PCa, using histopathology from ePLND as the reference standard. Additionally, we aimed to evaluate the interobserver reproducibility of MRI interpretation in this context.

## Study design

### Patients

This prospective multicenter observational study is based on 1,360 patients with newly diagnosed PCa between 2009 and 2016 in the county of Värmland, Sweden. To be eligible for inclusion, patients had to meet the following criteria: intermediate or high-risk PCa according to D`Amico classification, ASA physical status classification I–III, age ≤ 76 years, Gleason sum 7–9, clinical stage T1c-T3, PSA levels between 10 and 100 ng/mL, an estimated risk for N1 > 20% according to the Briganti nomogram and eligibility for curative treatment.

The exclusion criteria included contraindications for laparoscopy or MRI, previous local external beam radiotherapy (EBRT), a clinically estimated life expectancy of < 10 years, or confirmed distant metastases as determined by the axial skeleton MRI or bone scintigraphy.

Data were retrieved from patient’s electronic medical records and were also reported to the Swedish National Prostate Cancer Register.

In total, 99 patients met those criteria, Patient characteristics of the study cohort are summarized in Table 1. The study was approved by the local Ethics Committee, and informed consent was obtained from all participants (ethical approval numbers 2011 − 174 and 2016-11-28).

**Table 1:** Clinical and pathological characteristics

*N* = 99	
PSA (ng/mL) mean (SD)^a^	24 (19.5)
Age (years) mean (SD)^a^	67 (5.7)
Clinical stage (cT)	*n (%)*
T1c	15 (15.2)
T2	31 (31.3)
T3	53 (53.5)
Biopsy Gleason sum, n (%)	
6	7 (7.1)
7	64 (64.6)
8	16 (16.2)
9	12 (12.1)
Risk Group according to D`Amico criteria, n (%)	
Intermediate	7 (7.0)
High	92 (93.0)
N1 patients, *n* (%)	39 (39.4)
Lymph nodes per patient, *n* median (IQR^2^, min–max)	17 (8, 6–40)
Harvested lymph nodes, *n*	1,758
Histologically N1 lymph nodes, *n* (%)	155 (8.8)

^a^SD: Standard deviation

^b^IQR: Interquartile range

### MRI investigation

All patients underwent pelvic MRI before ePLND at one of the four participating centers. The MRI systems used are summarized in Table 2.

**Table 2:** MRI investigations/systems

	*N*, Primary mp MRI (*n*), Hospital	MRI system
**3.0 T**	**62 (50)**	
	29 (29), University Hospital	3 T Achieva, Philips Medical Systems, Best, The Netherlands
	33 (20), Central Hospital	3 T Siemens MAGNETOM Skyrafit
**1.5 T**	**34 (1)**	
	4 (n/a), Rural Hospital 1	1.5 T GE Optima 450w
	19 (n/a), Central Hospital	1.5 T Siemens Avantofit
	10 (n/a), Rural Hospital 2	1.5 T Philips Achieva dStream
	1 (1), Private Health Provider	1.5 T Philips Ingenia
**1.0 T**	3 (n/a)	
	3 (n/a), Central Hospital	1.0 T Siemens Harmoni Expert

mp = Multiparametric n/a = not applicable

In cases where a primary multiparametric (mp) MRI was performed, the pelvic lymph nodes were evaluated using a protocol that included axial T1W and T2W Turbo Spin Echo (TSE) sequences, with a slice thickness of 5 mm and an interslice gap of 6 mm.

For the remaining cases, the protocol consisted of axial and coronal T1W TSE sequences and axial and coronal T2W Short Tau Inversion Recovery (STIR) sequences for fat suppression, with a slice thickness of 5 mm, except for one MRI system which used 4 mm slices. The interslice gap varied depending on the MRI system and imaging plane, with values of 6.0 mm, 6.5 mm, 7.0 mm, or 7.5 mm. The field of view (FOV) also varied slightly between MRI systems. Nevertheless, all examinations covered the pelvic region distally from the sacral promontory.

The assessment of the lymph nodes was left to the discretion of each radiologist, who was responsible for identifying, quantifying, and localizing N1 disease. No DWI or contrast-enhanced sequences were used in the assessment. Radiologists were blinded to clinical information when performing the image evaluation. Interobserver analysis was conducted among three readers: Reader 1, an experienced radiologist at a university hospital; Reader 2, radiologists at a high-volume private center; and Reader 3, a radiologist at a rural hospital.

### Surgical technique

**Fig. 1:**
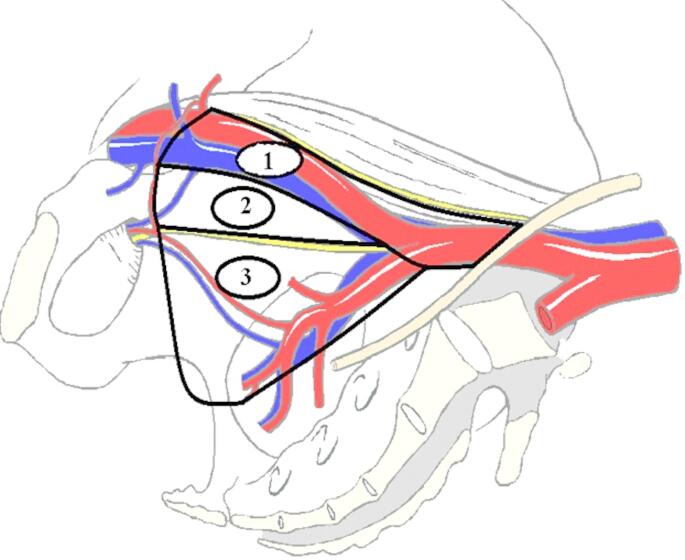
Extended pelvic lymph node dissection (ePLND) Sites: (1) External and common iliac vessels; (2) Obturator fossa; (3) Internal iliac vessels. *The diagram used with permission from the Department of Urology*,* Bern*,* Switzerland*

Laparoscopic ePLND was performed by four surgeons following a standardized technique as described by Mattei et al. [11]. Lymphatic tissue was harvested from the following anatomical spaces: Space (1) the external iliac vessels, which respect the genitofemoral nerve; Space (2) the obturator fossa, which is dissected between the external iliac vein and the obturator nerve; and Space (3) the remaining lymphatics under the obturator nerve and the internal iliac artery. The procedure was performed bilaterally (Fig. 1).

### Histopathological evaluation

The lymph node samples were categorized according to the anatomical spaces outlined in the surgical protocol. The lymph nodes were sectioned at 3 mm intervals, depending on their size. Immunohistochemistry was employed to confirm small metastases when necessary.

### Statistical analysis

The sample size calculation assumed that MRI has a sensitivity of 70% and that ePLND identifies all patients with N1 disease. To achieve 80% power and a 5% significance level, a minimum of 40 patients (20 per group) were needed [12, 13]. Given that local data suggest a 40% N1 incidence among patients undergoing ePLND, approximately 100 patients were included.

To evaluate the accuracy of the MRI-based classification, the sensitivity and specificity on a patient level were calculated, and pooled values were calculated via the R Shiny App MetaDTA [14]. Interobserver agreement among the three radiologists was assessed via Cohen’s κ for pairwise comparisons and Fleiss’ κ for overall concordance.

Group comparisons were performed via the Mann‒Whitney U test or Student’s t-test for continuous variables and the chi‒square test (or Fisher’s exact test) for categorical variables, with a two-tailed significance level set at *p* < 0.05. Values are presented as median interquartile range (IQR) for nonnormally distributed variables and as mean standard deviation (SD) for normally distributed variables. Categorical variables are reported as absolute and relative numbers. Data analysis was conducted via SPSS Statistics version 29 (IBM SPSS, New York, NY, USA).

## Results

Most of the 99 patients had a biopsy Gleason sum of 7 (64.6%) and were classified as having clinical stage T3 disease (53.5%). The majority (93.0%) were categorized as high-risk, with only 7.0% in the intermediate-risk group. The mean PSA level was 24 ng/mL (Table 1). A total of 1758 lymph nodes were harvested during ePLND, with a median of 17 (range 6–40) per patient. Among the patients, 39 (39.4%) were found to have N1 disease (Table 1).

The pooled sensitivity and specificity of T1W and T2W MRI sequences for N1 detection were 24.5% (95% CI: 16.3–35.1) and 95% (95% CI: 85.3–98.8), respectively. The sensitivity, specificity, and accuracy for each reader are detailed in Table 3. Interobserver agreement was moderate, with a Fleiss κ of 0.56 (95% CI: 0.44–0.67). The pairwise Cohens’ κ values ranged from 0.42 to 0.63, indicating moderate to substantial concordance.

**Table 3:** Diagnostic performance and interobserver analysis per patient

	Sensitivity (%)	Specificity (%)	Accuracy (%)
	(95% CI)	(95% CI)	(95% CI)
Reader 1	18.0 (7.5–33.5)	100 (94.0–100)	67.7 (57.5–76.7)
Reader 2	33.3 (19.1–50.2)	91.7 (81.6–97.2)	68.7 (58.6–77.6)
Reader 3	23.1 (11.1–39.3)	91.7 (81.6–97.2)	64.7 (54.4–74.0)

Concordance analysis	Kappa (95% CI)	Degree of Concordance	Agreement
Reader 1 * Reader 2	0.42 (0.18–0.67)	Moderate	86.9%
Reader 1 * Reader 3	0.63 (0.39–0.88)	Substantial	92.9%
Reader 2 * Reader 3	0.63 (0.42–0.84)	Substantial	89.9%

Reader agreement varied between 86.9% and 92.9%, with the highest agreement between readers 1 and 3 (Table 3). Overall, all three readers agreed in most cases (84.8%), with greater agreement for lymph node negative cases (88.3%) than for lymph node positive cases (79.5%). Only six patients were correctly classified as lymph node positive by all three readers, while three additional patients were correctly classified as lymph node positive by two readers and five by a single reader (Table 4). Notably, all readers failed to correctly identify 64,1% of the lymph node positive patients.

**Table 4:** Agreement between readers in patients with positive and negative lymph nodes

	Actual lymph node status	
	LN-, *n* (%)	LN+, *n* (%)
Classified as LN-		
- By all readers	53 (88.3)	25 (64.1)
- By two readers	0 (0)	0 (0)
- By one reader	0 (0)	0 (0)
Classified as LN+		
- By all readers	0 (0)	6 (15.4)
- By two readers	3 (5.0)	3 (7.7)
- By one reader	4 (6.7)	5 (12.8)
Total	60	39

LN- = negative lymph node and LN + = positive lymph node

The median interval from MRI to ePLND was 44 days (IQR 41). Notably, eight patients underwent surgery more than 100 days after their MRI. Of these, one was a true positive for N1 by all readers, two were true negatives, two were false positives, and three were false negatives.

A subgroup analysis of patients with N1 disease compared PSA levels, Gleason sums, and the number of metastatic lymph nodes between true-positive and false-negative MRI cases, creating two patient groups per reader (Table 6). No significant differences in PSA levels were found between true-positive and false-negative patients. Conversely, significant differences in Gleason sums were observed between the two readers (*p* = 0.018 and *p* = 0.028). Significant differences in the median number of metastatic lymph nodes were observed for Reader 1 (*p* = 0.034) and Reader 3 (*p* = 0.036) but not for Reader 2 (*p* = 0.63). All readers failed to identify patients with high metastatic burdens. Specifically, Reader 1 missed 11 patients with more than three metastatic lymph nodes (maximum of 12), Reader 2 missed 10 patients with more than three metastatic lymph nodes (maximum of 16), and Reader 3 missed 10 patients with more than three metastatic lymph nodes (maximum of 11). Furthermore, the identification of patients with a high number of metastatic lymph nodes was inconsistent across readers, as demonstrated in Table 5.

**Table 5:** Comparative analysis of the N1 group per reader

	Reader 1			Reader 2			Reader 3		
	MRI TP	MRI FN	*p* value	MRI TP	MRI FN	*p* value	MRI TP	MRI FN	*p* value
Patients, *n*	7	32	n/a	13	26	n/a	9	30	n/a
Nodal spread^a^*Median (IQR)* *Min–max*	6 (7) 1–16	2 (4) 1–12	0.034	3 (6.5) 1–12	2.5 (4) 1–16	0.63	6 (9) 1–16	2 (3) 1–11	0.036
PSA total ng/mL^a^*Median (IQR)*	35 (17)	21 (19.5)	0.16	28 (22)	21 (21)	0.31	35 (20)	19 (19.5)	0.054
Biopsy Gleason Sum^b^	7	7	0.06	7	7	0.018	7	7	0.028
7	42.9%	78.1%		53.8%	80.8%		44.4%	80.0%	
8	42.9%	9.4%		38.5%	3.8%		44.4%	6.7%	
9	14.3%	12.5%		7.7%	15.4%		11.1%	13.3%	

TP = True positive, FN = False negative, n/a = not applicable, IQR = InterQuartile Range

^a^Mann-Whitney U test ^b^ Fishers exact test

## Discussion

This study aimed to assess the performance of MRI in determining the lymph node stage of PCa patients based solely on morphological characteristics derived exclusively from T1W and T2W sequences, as current imaging protocols for the lymph node evaluation do not include DWI. To our knowledge, no recent studies have evaluated MRI without functional sequences in PCa staging, except for the Hovels meta-analysis (1980–2003) [5]. Our findings demonstrate a pooled sensitivity of 24.5% and a pooled specificity of 95%. Notably, all the readers failed to detect patients with high-volume metastatic disease.

Hovels reported a higher sensitivity (39%), which is likely attributable to suboptimal histological reference standards. In many cases, only limited PLND was performed, potentially leading to an underestimation of true N1 prevalence. Conversely, their lower specificity (82%) may reflect technological advancements in MRI over the years. Given that 1.5T MRI was introduced in the mid-1980s and that 3T MRI emerged between 1999 and 2002, Hovels findings may now be outdated. Additionally, studies included in the meta-analysis lacked standardized patient selection criteria, particularly regarding PCa risk stratification, PSA levels, clinical T stage, and biopsy-based Gleason scores.

In accordance with the Hovel findings, MRI demonstrated sensitivity comparable to that of CT (42%) while maintaining similar specificity. Consequently, our results can be compared with those of a recent nationwide Swedish registry study, which reported an 18% N1 detection rate for CT in high-risk PCa patients. This closely aligns with the MRI detection rates in our study (7%, 14%, and 18% for the three readers, respectively), yielding a pooled detection rate of 13% [15]. As the data were derived from the National Prostate Cancer Register of Sweden (NPCR) between 2014 and 2019, they reflect real-world clinical practice and support the generalizability of our findings.

### MRI with diffusion-weighted imaging (DWI)

Conventional MRI relies on size and morphological criteria for assessment. Given that over 80% of metastatic lymph nodes in PCa are less than 8 mm in size [16], detection rates remain low. The addition of functional imaging, such as DWI, has demonstrated superior performance [6]. A study utilizing 3T MRI with T1W, T2W, and DWI/ADC achieved a sensitivity of 55% and a specificity of 90% [17]. Similarly, Vallini et al. (2016) reported 84.6% sensitivity in intermediate- and high-risk PCa patients, using an ADC threshold value of 0.91 × 10^–3^ mm^2^/s, with ePLND as the reference standard [18]. However, the authors acknowledged the need for validation in larger studies and highlighted potential limitations, including DWI motion artifacts and the inability to detect micrometastases in lymph nodes smaller than 5 mm. Differentiating malignant from benign nodes via DWI and ADC values was superior to the use of size criteria alone, as demonstrated by Eiber et al. (2010) [7].

Conversely, a 2020 meta-review suggested that DWI provides only a modest improvement in diagnostic performance [19]. The review highlighted several limitations, including false-positive results due to necrotic or reactive nodes, partial volume effects in normal-sized lymph nodes, and artifacts from motion or bowel gas. Additionally, ADC values vary across MRI vendors, further complicating standardization.

### Alternative modalities

The 2024 European guidelines on metastatic staging highlight that choline and ^68^Gallium PSMA PET-CT, along with whole-body MRI, outperform traditional bone scans and CT in detecting metastatic disease [2]. However, this conclusion is largely based on the findings of Hofman´s proPSMA randomized multicenter trial [20], which included a mix of regional and distant metastases. A systematic review and meta-analysis by Perera et al. included only five studies with PLND as the reference for lymph node staging, likely overestimating PSMA PET‒CT sensitivity at 77% [21]. A recent meta-analysis suggested that PSMA PET-CT may outperform MRI, with pooled sensitivities of 65% and 41%, respectively, while maintaining comparable specificity [8]. However, the included studies were predominantly retrospective and varied in methodology. Interestingly, the reported MRI sensitivity in this meta-analysis closely mirrors that of the Hovels earlier study (39%) [5], which likely did not include DWI sequences. In contrast, a study on ^18^F-PSMA-PET/CT using strict methodology and ePLND as the histological reference showed a sensitivity of only 26.9% [22].

Despite some promising findings, high-level evidence supporting treatment decisions based on PSMA PET-CT remains limited, and its global availability is restricted [9]. Consequently, the routine use of PET-CT for initial staging is not yet recommended [23]. Given the widespread use of MRI for PCa detection prior to guided biopsies [24, 25], optimizing MRI for metastatic screening could be a cost-effective approach. This study emphasizes the need for standardized guidelines concerning the use of DWI and more advanced criteria for N1 evaluation. Future research should focus on incorporating functional imaging, potentially enhanced by Artificial Intelligence algorithms, to improve the differentiation of metastatic from benign lymph nodes. The authors conclude that combining DWI - without necessarily relying on ADC values - with morphological criteria from T1W and T2W sequences, might provide superior diagnostic performance compared to morphological assessment alone.

### Study strengths and limitations

The strengths of this study include the high proportion of high-risk PCa patients (93%) and the robust histological reference standard provided by ePLND. The histological reference is critical for validating radiological modalities, and local data indicate that approximately 40% of high-risk PCa patients undergoing ePLND have N1 disease, an observation confirmed by this study (Table 3). Additionally, the median number of lymph nodes harvested (17 per patient) reinforces the reliability of the histopathological assessment. Another strength is the study´s focus on primary PCa staging, avoiding the confounding effects of patients’ biochemical recurrence after curative intent, which is a limitation of other studies [26]. Furthermore, the interobserver agreement analysis demonstrated that MRI interpretation based solely on morphological criteria was relatively reproducible (Table 4).

However, the time interval between MRI and ePLND varied considerably, particularly among patients enrolled in earlier years, introducing a potential bias related to disease progression. Additionally, owing to the absence of standardized N1 criteria in clinical guidelines, readers rely on subjective judgment to classify lymph nodes, reflecting real-world clinical practice. Another limitation is the absence of data on the size of lymph node metastases in the histopathological reference, which could have provided further insights into detection challenges.

## Conclusion

MRI using only T1W and T2W sequences has demonstrated limited effectiveness in lymph node staging for intermediate- and high-risk prostate cancer patients. The method struggles to identify even high-volume metastatic disease, indicating significant limitations for clinical decision-making. Additionally, interobserver analysis shows only moderate agreement, highlighting the variability in interpreting MRI findings on the basis solely of morphological criteria. These results emphasize the need for improved imaging protocols or the integration of advanced techniques such as DWI to increase diagnostic accuracy.

## Data Availability

Anonymised datasets generated and/or analysed during the current study are securely stored by the corresponding author. Due to legal and ethical considerations under applicable data protection regulations, data sharing is not guaranteed.
